# Gastric adenocarcinoma of the fundic gland type: A review of the literature

**DOI:** 10.1002/jgh3.13014

**Published:** 2023-11-30

**Authors:** Zhiyong Zhai, Wei Hu, Zhaoyu Huang, Zemin Chen, Sicun Lu, Wei Gong

**Affiliations:** ^1^ Department of Gastroenterology, Shenzhen Hospital Southern Medical University Shenzhen China; ^2^ The Third School of Clinical Medicine Southern Medical University Guangzhou China

**Keywords:** chief cell, endoscopic mucosal resection, endoscopic submucosal dissection, gastric adenocarcinoma of the fundic gland type, magnifying endoscopy with narrow‐band imaging

## Abstract

**Background and Aim:**

Gastric adenocarcinoma of the fundic gland type (GA‐FG) is a newly described tumor entity but lacking consensus. This review summarizes the key features and controversies regarding this uncommon neoplasm.

**Methods:**

We reviewed studies on GA‐FG published in English from 2007 to 2021.

**Results:**

We found that 327 cases (340 lesions) have been reported. GA‐FG lesions originate from deep layers of the gastric mucosa, with the following characteristics on conventional white‐light endoscopy examination. These lesions, macroscopically identified as submucosal tumor‐like 0‐IIa, tend to have a whitish discoloration without inflammation, atrophy, or intestinal metaplasia in the background mucosa. Tumors located in the upper third of the stomach are usually solitary, with an average size <10 mm. Contrastingly, magnifying endoscopy with narrow‐band imaging mostly shows the absence of any demarcation line, with a regular microvascular pattern and regular microsurface pattern. GA‐FGs are covered with normal foveolar epithelium, forming a so‐called endless glands pattern in the deeper region, which are mainly composed of chief cells or parietal cells. Most tumors exhibit submucosal invasion, but lymphovascular invasion and nodal metastasis are rare. Regarding the treatment of GA‐FG, endoscopic submucosal dissection (ESD) and endoscopic mucosal resection (EMR) are effective treatment methods.

**Conclusions:**

GA‐FG is a rare tumor that typically follows a benign course. This neoplasm has distinct endoscopic and pathological features and could be treated by ESD or EMR.

## Introduction

Nakamura *et al*.^1^ grouped gastric adenocarcinoma into the differentiated and undifferentiated types based on the tumor's ability to form glands. It is widely accepted that differentiated adenocarcinomas that develop from intestinal metaplasia involving *Helicobacter pylori* (*H. pylori*) infection have an intestinal phenotype, whereas undifferentiated adenocarcinomas that develop from the gastric mucosa without intestinal metaplasia have a gastric phenotype. However, an immunohistochemical analysis of mucins has indicated that some differentiated adenocarcinomas have a gastric phenotype. Yao *et al*.^2^ reported cases of extremely well‐differentiated adenocarcinoma in 2006, similar to the foveolar epithelium, mucous neck cells, and pyloric glands. Tsukamoto *et al*.^3^ initially reported a case of gastric adenocarcinoma with chief cell differentiation in 2007. Furthermore, in 2010, Ueyama *et al*.^4^ reported 10 cases that showed differentiation to chief cells and proposed a new disease entity termed gastric adenocarcinoma of the fundic gland type (GA‐FG) based on their clinicopathological features. GA‐FGs are rarely observed clinically, and the majority of them are observed in Asia (South Korea and Japan). The first case was reported in China in 2018 and had obvious endoscopic and pathological features. The definition of this tumor is currently controversial. In view of its malignant characteristics, such as submucosal infiltration, submucosal stromal reaction, and lymphovascular invasion, the definition of GA‐FG is widely accepted. However, the tumor does not show significant progression or recurrence in the long‐term follow‐up before or after treatment; therefore, some scholars believe that the definition of an oxyntic gland polyp/adenoma is more appropriate.^5^ The 2019 World Health Organization (WHO) classification of gastrointestinal tumors recommends that cases with submucosal infiltration be termed GA‐FG while others are termed oxyntic gland polyps/adenoma. However, it is unknown whether GA‐FG develops from an oxyntic gland polyp/adenoma. According to the Japanese criteria for diagnosis, a tumor without submucosal invasion is considered the intramucosal stage of GA‐FG. Recently, researchers have reported a highly invasive variant of GA‐FG, called the gastric adenocarcinoma of the fundic gland mucosa type (GA‐FGM), which differentiates into gastric fundic gland and foveolar epithelium.^6^ Owing to the eradication treatment of *H. pylori* and the improvement of public health, reports on GA‐FGs have gradually increased in the recent years; however, there is a lack of common understanding of this tumor. Therefore, we conducted a review of studies on GA‐FG published in English language from 2007 to 2021.We found that 327 cases (340 lesions) have been reported so far, with the advances in biological characteristics and clinical management.

## Epidemiology

The incidence of GA‐FG is extremely low at 0.07% and 0.01%, as reported by Ueyama *et al*.^7^ and Tohda *et al*.,^8^ respectively. Park *et al*.^9^ investigated 6000 patients with gastric adenocarcinoma and found that only 3 cases (0.05%) were diagnosed with GA‐FG, whereas Miyazawa *et al*.^10^ reported only 5 cases (0.98%) of GA‐FG in 506 patients with gastric adenocarcinoma. The majority of GA‐FGs were observed in Japan and South Korea, whereas a study by Singhi *et al*.^5^ found that GA‐FGs were prevalent among 8 out of 10 non‐Asian individuals, including 4 Hispanics, 2 Caucasians, and 2 African Americans. Chen *et al*.^11^ also reported a case of a 79‐year‐old Caucasian male, which indicated that GA‐FG may occur worldwide. The patients were generally older adults, with an average age of 66.5 years (range, 38–91 years). There seemed to be a slight male predilection, with an M/F ratio of 1.7:1.

## Clinical presentation

The clinical characteristics of the 327 previously reported cases are presented in Table 1. GA‐FGs generally have no or mild subjective symptoms; however, Singhi *et al*.^5^ found that all 10 cases had clinical symptoms of gastroesophageal reflux, indicating that gastric acid secretion is maintained because of a lack of mucosal atrophy. Conventional gastric adenocarcinoma (CGA) is commonly accompanied by anemia, whereas 2 of 25 patients with GA‐FGs (8.0%) developed anemia. Of the 21 reported GA‐FGs, only 4 cases had a prior history of chronic gastritis and gastric ulcer, although 3 of 4 cases had been treated with *H. pylori* eradication therapy, suggesting that the etiology and pathogenesis of GA‐FG is different from those of CGA. Indeed, most GA‐FGs were free from *H. pylori* infection. For example, the prevalence of *H. pylori* in GA‐FGs was 11.7%, which is much lower than the 90% in CGA.^12^, ^13^

**Table 1:** Clinical characteristics of GA‐FG

Author (yr)	Cases (lesions)	Sex (M: F)	Age (yr, average)	Clinical symptoms	*H.pylori* (Pos: Neg: Era)	Treatment (ESD: EMR: OPE)	Survival time (mo, median)	Outcome
Iwamuro *et al*.^12^ (2021)	116 (126)	75:41	66.4 (46–91)	ND	13:38:60	72:42:3 ESD + OPE:2 Only biopsy:7	11 (0–107)	Alive, NED:112 Local recurrence:1 ND:13
Ueyama *et al*.^35^ (2021)	55	33:22	66.9 (38–87)	ND	5:28:9	45:6:4	1–113	Alive, NED:44 ND:11
Imamura *et al*.^34^ (2021)	14	4:10	66.9	ND	0:11:3	ND	ND	ND:14
Ushiku *et al*.^6^ (2020)	14	9:5	60.8 (41–85)	ND	0:14:8	13:0:0 NEWS:1	3–97	Alive, NED
Li *et al*.^50^ (2020)	8	3:5	65.0 (48–80)	Reflux:5 Abdominal pain and distention:3	0:8:0	8:0:0	17(5–33)	Alive, NED
Fan *et al*.^51^ (2020)	1	1:0	60	ND	1:0:0	1:0:0	18	Alive, NED
Peng *et al*.^52^ (2020)	1	0:1	60	ND	0:1:0	1:0:0	ND	ND
Chen *et al*.^17^ (2019)	1 (2)	0:1	55	Distention	0:0:1	2:0:0	3	Alive, NED
Ishibashi *et al*.^26^ (2019)	8	4:4	71.6 (51–83)	ND	0:4:4	8:0:0	18–48	Alive, NED
Uozumi *et al*.^49^ (2019)	1	1:0	73	ND	ND	1:0:0	3	Alive, NED
Kino *et al*.^18^ (2018)	1 (2)	1:0	78	ND	0:0:1	2:0:0	12	Alive, NED
Okumura *et al*.^32^ (2018)	1	1:0	55	ND	ND	0:0:1	ND	ND
Kai *et al*.^24^ (2018)	1	0:1	76	Abdominal pain	1:0:0	1:0:0	ND	ND
Imagaw *et al*.^16^ (2018)	1	1:0	73	ND	0:0:1	ND	ND	ND
Komeda *et al*.^53^ (2017)	1	1:0	50	Mild anemia	0:1:0	0:1:0	ND	ND
Manabe *et al.* ^54^ (2017)	1	1:0	58	ND	0:0:1	ND	ND	ND
Chiba *et al*.^23^ (2016)	20	16:4	68.8 (44–85)	ND	13:5:2	9:0:0 Only biopsy:11	ND	ND
Tohda *et al*.^8^ (2016)	4	2:2	53.5 (42–62)	ND	2:2:0	4:0:0	ND	ND
Sato *et al*.^14^ (2016)	1	0:1	77	No	0:1:0	1:0:0	48	Alive, NED
Miyazawa *et al*.^10^ (2015)	5	3:2	72.2 (67–78)	ND	0:5:0	4:0:0 ESD + OPE:1	ND	ND
Ota *et al*.^25^ (2015)	1	0:1	69	ND	0:0:1	ND	ND	ND
Parikh *et al*.^55^ (2015)	1	1:0	66	Mild anemia	0:1:0	0:1:0	ND	ND
Kato *et al*.^48^ (2015)	1	1:0	80	ND	0:1:0	CLEAN‐NET	ND	ND
Takeda *et al*.^15^ (2015)	1	1:0	66	ND	0:0:1	1:0:0	ND	ND
Hori *et al*.^56^ (2015)	1	1:0	79	ND	ND	1:0:0	12	Alive, NED
Nomura *et al*.^19^ (2014)	26	22:4	66 (49–79)	ND	0:1:0	19:0:7	ND	ND
Ueo *et al*.^33^ (2014)	1	1:0	62	ND	3:6:1	ESD + OPE	ND	ND
Ueyama *et al*.^7^ (2013)	10	6:4	66.5 (55–78)	ND	ND	7:2:0 ESD + OPE:1	1–19	Alive, NED
Kushima *et al*.^57^ (2013)	3	1:2	68 (56–78)	ND	0:1:0	3:0:0	6–63	Alive, NED
Abe *et al*. (2013)	1	0:1	71	ND	ND	0:1:0	12	Alive, NED
Singhi *et al*.^5^ (2012)	10	4:6	67.2 (44–79)	Reflux	ND	Polypectomy:9 ND:1	6–39	Alive, NED:9 ND:1
Park *et al*.^9^ (2012)	3	3:0	65 (47–76)	ND	ND	1:0:1 ESD + OPE:1	11–32	Alive, NED
Chen *et al*.^11^ (2012)	1	1:0	79	GERD	ND	0:1:0	2	Alive, NED
Terada *et al*.^58^ (2011)	1	1:0	78	Abdominal pain	ND	Only biopsy	ND	Died (unknown)
Fukatsu *et al*.^59^ (2011)	1	1:0	56	ND	ND	0:1:0	ND	ND
Ueyama *et al*.^4^ (2010)	10	6:4	65.0 (42–79)	ND	ND	5:2:3	27 (10–70)	ND
Tsukamoto *et al*.^3^ (2007)	1	0:1	82	ND	ND	0:1:0	ND	ND

CLEAN‐NET, combined laparoscopic and endoscopic approach for neoplasia with a non‐exposure technique; EMR, endoscopic mucosal resection; Era, eradication; ESD, endoscopic submucosal dissection; F, female; GERD, gastroesophageal reflux disease; *H. pylori, Helicobacter pylori*; M, male; mo, month; ND, not described; NED, no evidence of disease; Neg, negative; NEWS, non‐exposed endoscopic wall‐inversion surgery; OPE, operation; Pos, positive; yr, year.

## Endoscopy and gross appearance

**Figure 1:**
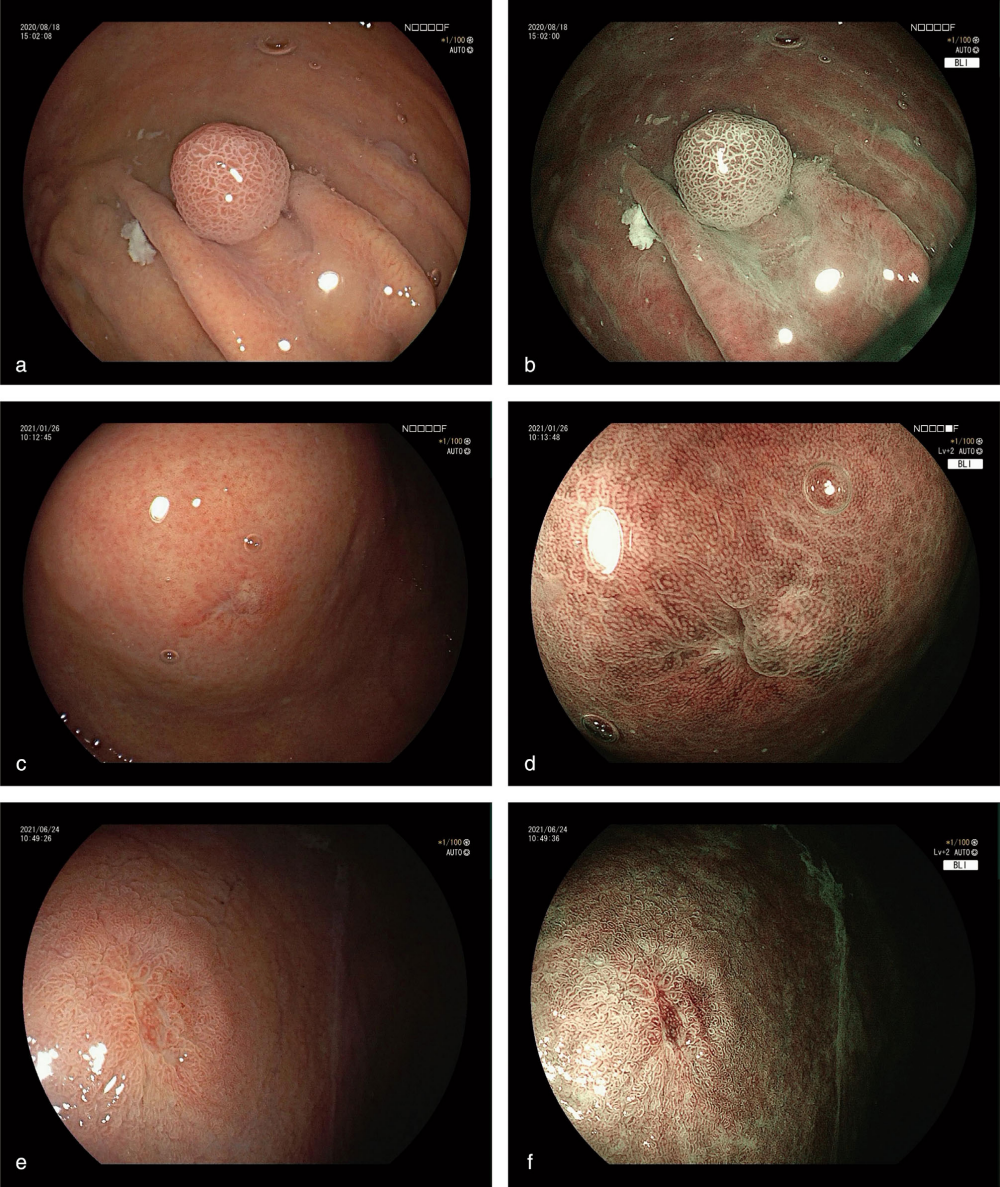
Representative images of white‐light imaging (WLI) and magnifying endoscopy with narrow‐band imaging (M‐NBI) findings. WLI showed 0‐1Ia type lesions with red or yellow color tone (a, c, e). M‐NBI showed no demarcation line, but showed regular or irregular microvascular pattern and microstructral pattern (b, d, f).

The endoscopic characteristics of 340 previously reported lesions are summarized in Table 2. These lesions, originating from deep layers of the gastric mucosa, were macroscopically identified as submucosal tumor (SMT)‐like type mainly, although an increasing number of lesions were of the flat/depressed type. Based on the Paris endoscopic classification, 216 of the 327 lesions (66.1%) showed a 0‐IIa type. Overall, 17 of 216 lesions (7.9%) were accompanied by central depression, which was believed to be evidence of biopsy scarring or submucosal involvement. In previous studies, the color tone of 203 lesions was examined, which appeared white in 107, normal in 50, and red or yellow in 46. Notably, some cases have documented black or brown pigmentation in the lesion,^12^, ^14^, ^15^, ^16^ which is taken as one of the endoscopic features of GA‐FG. This was considered to be the result of hemosiderin deposition due to tumor vascular disruption; however, further evidence is required. The vast majority of lesions, 254 out of 321 (79.1%), occurred in the upper third of the stomach. Notably, 66 out of 321 (20.6%) tumors developed in the middle third, and only 1 tumor was seen in the lower third, which was a significant difference from CGA which shows a predisposition to occur in the gastric antrum. Most cases had a single lesion, and several cases showed multiple lesions.^12^, ^17^, ^18^, ^19^ Typically, these lesions were small, with an average size of <10 mm, but the largest lesion measured 85 mm. Additionally, lymphovascular invasion was also observed in the largest lesion, and multiple gene mutations may be associated with florid aggressiveness. The above characteristics of GA‐FG can be observed on conventional white‐light endoscopy (WLI) examination (Fig. 1a, c, e) and a common consensus has been formulated; however, it is also easily missed in diagnosis and misdiagnosis.

**Table 2:** Endoscopic characteristics of GA‐FG

Author (yr)	Cases (lesions)	Location (U:M: L)	Size (mm, average)	Macroscopic shape (0‐IIa: 0‐IIb/IIc)	Color tone (white: yellow: red: normal)	M‐NBI			
						Vasodilatation (+:–)	DL (+:–)	MVP (regular: irregular: absent)	MSP (regular: irregular: absent)
Iwamuro *et al*.^12^ (2021)	116 (126)	93:33:0	6.2 (2–25)	76:48 0‐Ia:2	56:7:14:49	81:45	ND	ND	ND
Ueyama *et al*.^35^ (2021)	55	46:9:0	8.4 (1.5–43)	37:18	ND	ND	ND	ND	ND
Imamura *et al*.^34^ (2021)	13 (14)	12:0:0 Remnant:2	5.1 (1–9)	11:3	8:0:5:1	12:2	0:14	13:1:0	14:0:0
Ushiku *et al*.^6^ (2020)	14	ND	7.4 (3–15)	12:2	ND	12:2	ND	14:0:0	ND
Li *et al*.^50^ (2020)	8	7:1:0	6.0 (4–12)	6:2	ND	ND	1:7	4:4:0	ND
Fan *et al*.^51^ (2020)	1	0:1:0	12	0:1	1:0:0:0	1:0	ND	1:0:0	1:0:0
Peng *et al*.^52^ (2020)	1	1:0:0	8	1:0	0:0:1:0	1:0	1:0	0:1:0	0:1:0
Chen *et al*.^17^ (2019)	1 (2)	1:1:0	5.0 (4–6)	1:1	2:0:0:0	ND	ND	1:1:0	ND
Ishibashi *et al*.^26^ (2019)	8	6:2:0	7 (4–14)	6:2	8:0:0:0	8:0	3:5	8:0:0	5:1:2
Uozumi *et al*.^49^ (2019)	1	1:0:0	25	1:0	ND	ND	0:1	1:0:0	1:0:0
Kino *et al*.^18^ (2018)	1 (2)	2:0:0	5.5 (4–7)	2:0	0:2:0:0	2:0	ND	2:0:0	ND
Okumura *et al*.^32^ (2018)	1	0:1:0	47	1:0	1:0:0:0	ND	ND	ND	ND
Kai *et al*.^24^ (2018)	1	0:1:0	28	0:1	ND	ND	1:0	ND	0:1:0
Imagaw *et al*.^16^ (2018)	1	1:0:0	4	1:0	ND	1:0	0:1	ND	ND
Komeda *et al*.^53^ (2017)	1	1:0:0	5	1:0	0:0:1:0	ND	0:1	0:1:0	ND
Manabe *et al*.^54^ (2017)	1	1:0:0	10	1:0	ND	ND	ND	1:0:0	1:0:0
Chiba *et al*.^23^ (2016)	20	14:6:0	ND	12:8	11:9:0:0	ND	ND	4:16:0	ND
Tohda *et al*.^8^ (2016)	4	3:1:0	4.0 (2–5)	2:2	4:0:0:0	4:0	ND	4:0:0	4:0:0
Sato *et al*.^14^ (2016)	1	1:0:0	10	1:0	0:0:1:0	1:0	0:1	1:0:0	1:0:0
Miyazawa *et al*.^10^ (2015)	5	5:0:0	7.8 (5–13)	4:1	5:0:0:0	ND	ND	3:2:0	3:0:2
Ota *et al*.^25^ (2015)	1	ND	ND	ND	1:0:0:0	ND	ND	ND	ND
Parikh *et al*.^55^ (2015)	1	1:0:0	7	0‐Ia	0:0:1:0	ND	ND	0:1:0	ND
Kato *et al*.^48^ (2015)	1	ND	15	1:0	1:0:0:0	ND	0:1	1:0:0	1:0:0
Takeda *et al*.^15^ (2015)	1	1:0:0	ND	0:1	0:0:1:0	ND	0:1	1:0:0	0:1:0
Hori *et al*.^56^ (2015)	1	0:1:0	1.6	1	1:0:0:0	ND	ND	ND	ND
Nomura *et al*.^19^ (2014)	26	23:3:0	15.3 (3–85)	21:5	ND	ND	ND	ND	ND
Ueo *et al*.^33^ (2014)	1	ND	44	0:1	ND	ND	1:0	0:1:0	1:0:0
Ueyama *et al*.^7^ (2013)	10	6:4:0	9.3 (3–31)	6:4	8:0:2:0	ND	0:10	10:0:0	ND
Kushima *et al*.^57^ (2013)	3	3:0:0	5.0 (3–8)	0‐Ia:3	ND	ND	ND	ND	ND
Abe *et al*. (2013)	1	1:0:0	ND	1:0	0:1:0:0	ND	ND	ND	ND
Singhi *et al*.^5^ (2012)	10	10:0:0	4.0 (2–8)	ND	ND	5:5	ND	ND	ND
Park *et al*.^9^ (2012)	3	1:1:1	2.6 (1.2–3.6)	3:0	ND	ND	3:0	ND	ND
Chen *et al*.^11^ (2012)	1	1:0:0	20	ND	ND	ND	1:0	ND	ND
Terada *et al*.^58^ (2011)	1	0:1:0	15	ND	ND	ND	ND	ND	ND
Fukatsu *et al*.^59^ (2011)	1	1:0:0	5	1:0	0:1:0:0	ND	ND	ND	ND
Ueyama *et al*.^4^ (2010)	10	10:0:0	8.6 (4–20)	5:5	ND	ND	10:0	ND	ND
Tsukamoto *et al*.^3^ (2007)	1	1:0:0	1.6	1:0	ND	ND	ND	ND	ND

DL, demarcation line; L, lower third; M, middle third; M‐NBI, magnifying endoscopy with narrow‐band imaging; MSP, microsurface pattern; MVP, microvascular pattern; ND, not described; U, upper third; yr., year.

In contrast, magnifying endoscopy with narrow‐band imaging (M‐NBI), a novel endoscopic imaging technology, is useful for the diagnosis of early gastric cancer. The Japanese Gastroenterological Association (JGA), Japan Gastroenterological Endoscopy Society (JGES), Japanese Gastric Cancer Association (JGCA), and World Endoscopy Organization (WEO) jointly devised a magnifying endoscopy simple diagnostic algorithm (MESDA) for gastric cancer based on the vessel plus surface classification system (VSCS) proposed by Yao *et al*.^20^, ^21^ The presence of a demarcation line (DL), an irregular microvascular pattern (MVP), and a microsurface pattern (MSP), which are specific features of early gastric adenocarcinoma, may not be found in GA‐FG. Notably, 41 of 62 lesions (66.1%) were devoid of DL, while 36 of 40 (90.0%) showed regular MVP, and 69 of 97 (71.1%) showed regular MSP (Fig. 1b, d, f). Matsumoto *et al*.^22^ reported that 25 of 48 M‐NBI diagnostic limitation lesions (M‐NBI‐DLLs) were diagnosed as GA‐FG, the reason for which is that GA‐FG is not exposed on the surface, further confirming its difference from CGA. Of 169 lesions, 120 (71.0%) presented with vasodilatation and branched vessels on the tumor surface, which is another characteristic feature.

**Figure 2:**
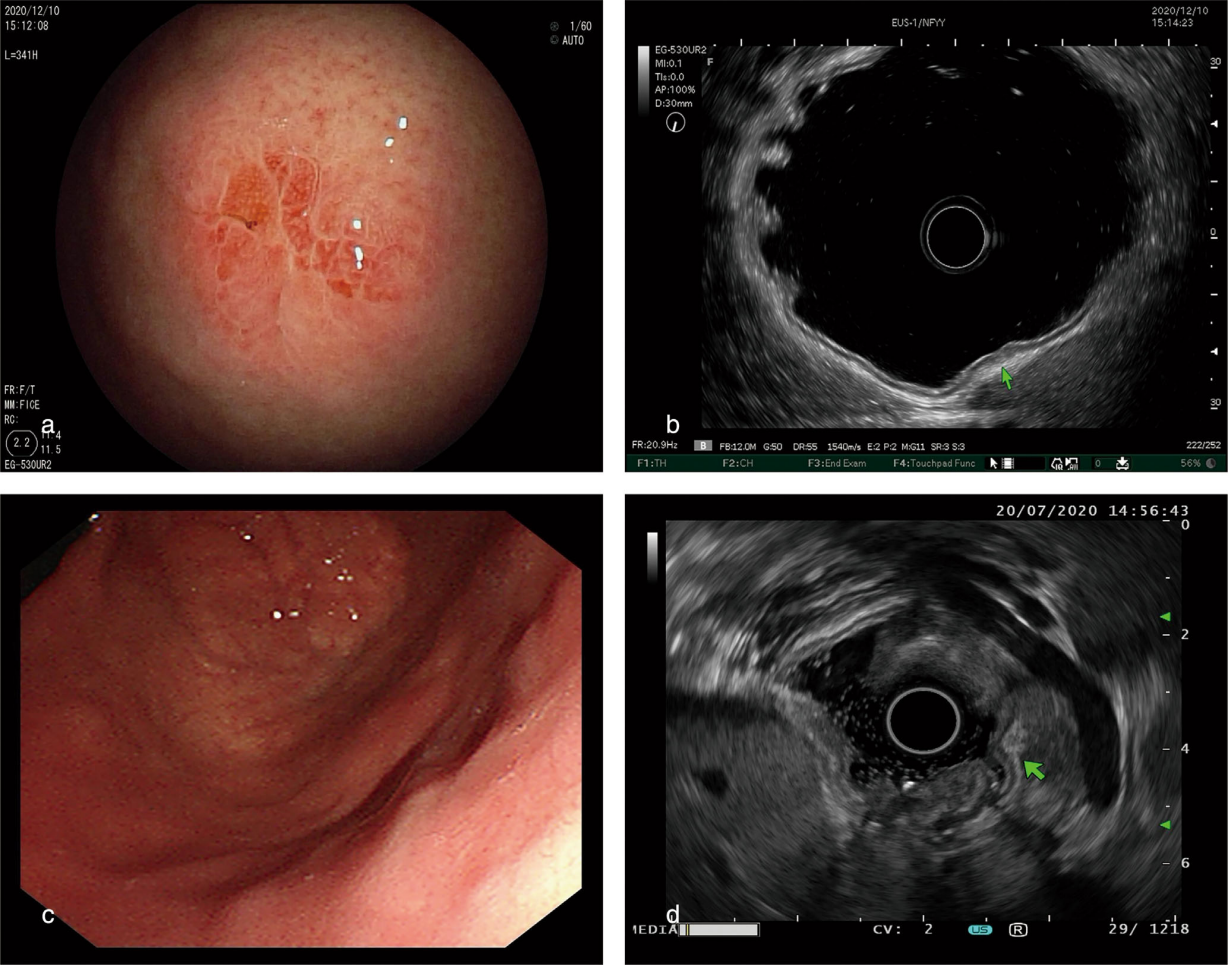
Representative images of white‐light imaging (WLI) and endoscopic ultrasound (EUS) findings. WLI showed SMT‐like type lesions (a, c). EUS showed lesions located in the deep layer of gastric mucosa or gastric submucosa, with hypoecho (b, d).

Endoscopic ultrasound (EUS), a combination of both endoscopy and ultrasound, which can not only show the surface of gastrointestinal mucosa but also the structure of the gastrointestinal wall and the relationship with adjacent organs, is helpful in confirming the origin, nature, and depth of invasion of GA‐FG (Fig. 2). Because GA‐FG, which originates from the deep layers of the mucosa, has a tendency to invade the submucosa, EUS combined with M‐NBI is used to diagnose this tumor.

## Histopathological findings

The histopathological characteristics of 340 previously reported GF‐FG lesions are presented in Table 3. An independent histologic assessment detected no background atrophy in nearly all GA‐FG lesions. Although the percentage of *H. pylori* positivity reported in one study reached a high level, 15 of 20 (75.0%) cases were positive for *H. pylori* with global atrophy, and the lesions developed in areas with no apparent background atrophy.^23^ However, inflammation or intestinal metaplasia was observed in the background mucosa of three lesions in another study.^9^ Most lesions were covered with a normal foveolar epithelium. Several lesions that were negative for MUC5AC, as shown by immunohistochemistry (IHC), showed foveolar epithelial involvement, which was considered to be partially transformed to GA‐FGM. The deeper region usually showed a mixture of clustered glands, expanded glands, and irregular anastomosing cords, producing a so‐called endless glands pattern, whereas cribriforming and glandular infolding was rarely seen. Some researchers believe that gland expansion may be due to the overexpression of aquaporin 4 (AQP4) in tumor cells, resulting in an excessive water inflow.^14^ The tumor glands exhibited marked atypia; however, prominent atypia and mitotic figures were not observed in the tumor cells. Only one lesion showed obvious interstitial reaction,^4^ while the others did not. Most GA‐FG lesions had a gastric phenotype, immunohistochemically negative for CD10, MUC2, and CD‐X2. However, Ueyama *et al*.^7^ reported that two lesions had a gastrointestinal phenotype, immunohistochemically positive for CD10. Although Kai *et al*.^24^ also found that IHC for CD10 was positive in one lesion; it was confirmed to be GA‐FG with a signet ring cell carcinoma component. All GA‐FG lesions were immunohistochemically positive for at least one of the following markers: pepsinogen‐I, MUC‐6, MUC5AC, and H+/K+ ‐ATPase. Based on the histopathological characteristics of GA‐FG reported in previous literature, GA‐FG is typically classified into three categories: the chief‐cell‐predominant type, the parietal‐cell‐predominant type, and the mixed type. The histopathological types of 169 lesions reported since 2007 were confirmed, with 161 (95.0%) in the chief‐cell‐predominant type and 8 in the mixed type. The chief‐cell‐predominant type is strongly positive for pepsinogen‐I and MUC‐6 and negative or weakly positive for H+/K+ ‐ ATPase immunohistochemically. It is primarily composed of well‐differentiated chief cells with a faintly basophilic cytoplasm and slightly enlarged basal nuclei; however, in some lesions of this type the tumor cells contain coarsely granular eosinophilic cytoplasm mimicking parietal cells.^6^, ^9^ An article published in 2015 suggested that chief cells are transdifferentiated from mucous neck cells, characterized by an IHC marker of MUC6, which is supposed to be lost in the process of transdifferentiation. In fact, the chief cells of GA‐FG retain the IHC marker MUC6; therefore, they are naïve.^25^ Although several lesions were immunohistochemically positive for MUC5AC, showing an additional differentiation to foveolar epithelium, they were still covered with the normal foveolar epithelium and were defined as GA‐FG.^26^ Gastric adenocarcinoma with parietal cell differentiation had been reported in the literature before 2007; however, the evidence of parietal cell differentiation in most of these studies is not conclusive.^27^, ^28^ In 2003, Yang *et al*.^29^ identified a case of parietal cell carcinoma of the gastric cardia, and the tumor cells were strongly positive for H+/K+ ‐ATPase immunohistochemically and had eosinophilic finely granular cytoplasm and hyperchromatic pleomorphic nuclei, which was probably a case of GA‐FG with parietal cell differentiation. A mixed type of GA‐FG, strongly positive for pepsinogen‐I, MUC‐6, and H+/K+ ‐ATPase immunohistochemically, shows an admixture of chief and parietal cells. Singhi *et al*.^5^ reported three lesions showing predominantly mucous neck cells, which may represent a fourth category, strongly positive for MUC‐6 and weakly positive for pepsinogen‐I and H+/K+ ‐ ATPase immunohistochemically; however, more evidence is needed to support this view.

**Table 3:** Histopathological characteristics of GF‐FG

Author (yr)	Cases (lesions)	BM (+:–)	Depth (M:SM) (μm, average)	Lympho‐vascular invasion (+:–)	PG‐I (+:–)	MUC6 (+:–)	H+/K+‐ATPase (+:–)	MUC5AC (+:–)	CD10 (+:–)	MUC2 (+:–)	p53 (+:–)	Ki‐67 (average)
Iwamuro *et al*.^12^ (2021)	116 (126)	ND	51:56 ND:19	2:124	67:4 ND:55	71:11 ND:44	24:15 ND:87	9:64 ND:53	0:15 ND:111	0:23 ND:103	ND	ND
Ueyama *et al*.^35^ (2021)	55	ND	0:55 (251.5)	1:54	55:0	54:1	41:8 ND:6	0:55	0:51 ND:4	ND	1:50 ND:4	4.7%:47 ND:8
Imamura *et al*.^34^ (2021)	13 (14)	0:14	5:9	0:14	ND	ND	ND	ND	ND	ND	ND	ND
Ushiku *et al*.^6^ (2020)	14	0:14	0:14 (429.3)	0:14	ND	ND	ND	ND	ND	ND	ND	ND
Li *et al*.^50^ (2020)	8	1:7	2:6 (181.7)	0:8	8:0	8:0	ND	ND	ND	0:8	0:8	<2%
Fan *et al*.^51^ (2020)	1	0:1	ND	ND	1:0	1:0	0:1	0:1	ND	0:1	0:1	<5%
Peng *et al*.^52^ (2020)	1	ND	M	0:1	1:0	1:0	1:0	0:1	0:1	0:1	0:1	1%
Chen *et al*.^17^ (2019)	1 (2)	0:1	2:0	0:2	2:0	2:0	2:0	0:2	0:2	0:2	ND	ND
Ishibashi *et al*.^26^ (2019)	8	4:4	5:3 (45.3)	0:8	8:0	8:0	2:6	8:0	ND	0:8	0:8	1–5%
Uozumi *et al*.^49^ (2019)	1	1:0	ND	0:1	1:0	1:0	ND	1:0	ND	ND	ND	ND
Kino *et al*.^18^ (2018)	1 (2)	0:2	1:1 (120)	0:2	2:0	2:0	2:0	0:2	ND	0:2	ND	ND
Okumura *et al*.^32^ (2018)	1	0:1	Subserosa	1:0	1:0	1:0	1:0	1:0	ND	ND	0:1	5%
Kai *et al*.^24^ (2018)	1	1:0	SM (400)	0:1	1:0	1:0	1:0	1:0	ND	1:0	ND	ND
Imagaw *et al*.^16^ (2018)	1	ND	ND	ND	ND	ND	ND	ND	ND	ND	ND	ND
Komeda *et al*.^53^ (2017)	1	ND	SM	ND	1:0	1:0	0:1	ND	ND	ND	ND	ND
Manabe *et al*.^54^ (2017)	1	ND	SM (450)	0:1	1:0	1:0	1:0	0:1	ND	0:1	ND	ND
Chiba *et al*.^23^ (2016)	20	17:3	2:8 (199.4)	2:7 ND:11	20:0	17:3	11:9	ND	ND	ND	ND	3.2%:9 ND:1
Tohda *et al*.^8^ (2016)	4	0:4	2:2	0:4	4:0	4:0	0:4	0:4	0:4	0:4	ND	<5%
Sato *et al*.^14^ (2016)	1	ND	SM (300)	1:0	ND	ND	ND	ND	ND	ND	ND	ND
Miyazawa *et al*.^10^ (2015)	5	0:5	0:5 (620)	1:4	5:0	5:0	ND	0:5	ND	0:5	0:5	<5%
Ota *et al*.^25^ (2015)	1	0:1	ND	ND	1:0	1:0	1:0	ND	ND	ND	ND	ND
Parikh *et al*.^55^ (2015)	1	ND	0:1	1:0	1:0	1:0	1:0	1:0	ND	ND	ND	ND
Kato *et al*.^48^ (2015)	1	1:0	SM (300)	0:1	1:0	1:0	1:0	ND	ND	ND	ND	ND
Takeda *et al*.^15^ (2015)	1	ND	ND	0:1	1:0	1:0	1:0	ND	ND	ND	ND	ND
Hori *et al*.^56^ (2015)	1	ND	M	0:1	1:0	1:0	1:0	0:1	0:1	0:1	ND	ND
Nomura *et al*.^19^ (2014)	26	ND	0:26 (417)	5:21	ND	ND	ND	ND	ND	ND	ND	ND
Ueo *et al*.^33^ (2014)	1	1:0	Subserosa	1:0	1:0	ND	ND	ND	ND	ND	ND	ND
Ueyama *et al*.^7^ (2013)	10	1:9	5:5 (360)	1:9	10:0	10:0	10:0	4:6	2:8	0:10	0:10	8.64%
Kushima *et al*.^57^ (2013)	3	0:1	1:2 (500)	ND	3:0	3:0	3:0	ND	ND	ND	ND	ND
Abe *et al*. (2013)	1	0:1	ND	0:1	1:0	0:1	0:1	0:1	0:1	0:1	0:1	1.9%
Singhi *et al*.^5^ (2012)	10	ND	10:0	10:0	ND	10:10	ND	0:10	ND	0:10	0:9 ND:1	2.6%
Park *et al*.^9^ (2012)	3	3:0	1:2 (201)	0:3	3:0	3:0	0:3	3:0	0:3	0:3	ND	ND
Chen *et al*.^11^ (2012)	1	0:1	SM	0:1	ND	ND	ND	ND	ND	ND	0:1	3.8%
Terada *et al*.^58^ (2011)	1	ND	ND	ND	ND	ND	ND	ND	ND	ND	ND	ND
Fukatsu *et al*.^59^ (2011)	1	ND	SM (100)	0:1	1:0	1:0	1:0	0:1	ND	ND	ND	ND
Ueyama *et al*.^4^ (2010)	10	1:7 ND:2	1:9 (844)	0:10	10:0	10:0	4:6	1:9	0:10	0:10	0:10	3.6%
Tsukamoto *et al*.^3^ (2007)	1	ND	ND	ND	1:0	1:0	0:1	ND	ND	ND	ND	7.9%

BM, background mucosa; M, mucosa; ND, not described; PG‐I, pepsinogen I; SM, submucosa; yr., year.

The majority of GA‐FG lesions, 210 of 300 (70.0%), exhibited submucosal invasion, with an average depth of 141 lesions of 348.0 μm (range, 2–4000 μm); however, observations of lymphovascular invasion and nodal metastasis are rare. IHC revealed that only 1 of 106 lesions (0.9%) was positive for p53, and the mean Ki‐67 index of 117 lesions was less than 4.5% (range, 0.2–20%), which is well below the positive rate of p53 (42.1%) and the mean Ki‐67 index (22.0%) in CGA.^30^, ^31^ GA‐FG is generally considered to have a low potential for malignancy, although two extremely rare lesions of advanced GA‐FG, one of which was negative for p53 and had a Ki‐67 index of 5% immunohistochemically, invaded the subserosa with lymphovascular invasion and nodal metastasis.^32^, ^33^ Given the malignant features of GA‐FG, the 2019 WHO classification of gastrointestinal tumors recommends that the presence of submucosal invasion in the lesion is the key feature to distinguish GA‐FG from oxyntic gland polyp/adenoma, which is also supported by most researchers.

GA‐FGM, which differentiates between the gastric fundic gland and foveolar epithelium, is considered to be a subtype of GA‐FG. Notably, 47 cases of GA‐FGM (47 lesions) have been reported in the literature, and the clinicopathological characteristics are summarized in Table 4. The deeper parts of the lesions covered with atypical foveolar epithelium showed complicated glands consisting of tumor cells with prominent atypia and were immunohistochemically positive for MUC5AC.^6^, ^34^, ^35^, ^36^, ^37^, ^38^, ^39^, ^40^ The average size of GA‐FGM was mostly >15 mm, and three lesions reported by Ushiku *et al*.^6^ even reached an average size of 26.8 mm. GA‐FGM lesions also have a tendency to invade the submucosa, with an average depth of more than 1000 μm. In terms of lymphovascular invasion and nodal metastasis in GA‐FGM lesions, Ueyama *et al*.^35^ reported that 6 of 25 lesions (24.0%) presented with lymphatic invasion and 5 of 25 lesions (20.0%) presented with vascular invasion, which were all significantly higher than the lesions of GA‐FG (*P* < 0.01, *P* < 0.05); however, only 1 of 13 lesions (7.7%) presented with nodal metastasis, which was similar to the lesions of GA‐FG (*P* = 0.26). Immunohistochemically, 4 of the 19 lesions (21.1%) were positive for p53, and the mean Ki‐67 index of 21 lesions was 9.9%, suggesting that GA‐FGM is more malignant than GA‐FG.

**Table 4:** Clinicopathological characteristics of GA‐FGM

Author(yr)	Cases	Size (mm, average)	Depth (M:SM) (μm, average)	Lymphovascular invasion (+:–)	MUC5AC (+:–)	p53 (+:–)	Ki‐67 (average)	Treatment (ESD: EMR: OPE)	Survival time (mo, median)	Outcome
Ueyama *et al*.^35^ (2021)	25	20.8 (4–85)	4:21 (1212.5)	Lymphatic: 6:19 Vascular: 5:20	25:0	4:15 ND:6	9.9%:21 ND:4	15:2:8	1–30	NED
Imamura *et al*.^34^ (2021)	12	7.5 (4–15)	4:8	0:12	ND	ND	ND	ND	ND	ND
Sato *et al*.^36^ (2021)	1	10	0:1 (1000)	0:1	1:0	ND	ND	1:0:0	ND	ND
Takahashi *et al*.^37^ (2021)	1	ND	1:0	0:1	1:0	ND	ND	1:0:0	ND	ND
Ushiku *et al*.^6^ (2020)	4	26.8 (17–40)	0:4 (2212.5)	2:2	ND	ND	ND	0:0:2 ESD + OPE:2	3–97	NED
Takahashi *et al*.^39^ (2020)	2	16.5 (8–25)	1:1 (400)	0:1	2:0	ND	ND	2:0:0	ND	ND
Uchida *et al*.^40^ (2018)	1	20	0:1	ND	1:0	1:0	14.0%	ESD + OPE	1	NED
Takahashi *et al*.^38^ (2017)	1	3	1:0	0:1	1:0	ND	ND	1:0:0	ND	ND

EMR, endoscopic mucosal resection; ESD, endoscopic submucosal dissection; M, mucosa; mo: month; ND, not discribed; NED, no evidence of disease; OPE, operation; SM, submucosa.

## Etiopathogenesis

However, the pathogenesis of GA‐FG remains unclear. Cases with *H. pylori* negativity or eradication account for the majority of GA‐FGs; hence, some researchers believe that the administration of proton pump inhibitors (PPIs) may be associated with the occurrence of GA‐FG, which can lead to chief‐cell and parietal‐cell hyperplasia.^41^, ^42^, ^43^ However, only 94 of 251 (37.5%) patients consumed this drug.

One study showed that nuclear β‐catenin immunolabeling was higher in GA‐FG (labeling index [LI]: median, 19.3%; high expression [LI > 30%], 25.9%) than in CGA invading the submucosa (CGA‐SM) (median LI, 14.7%; high expression, 5.3%). A genome sequencing analysis revealed that 14 of 27 GA‐FGs (51.9%) and 5 of 19 CGA‐SM cases (26.3%) harbored at least one gene mutation of *CTNNB1, AXINs,* and *APC*, suggesting that the Wnt/β‐catenin signaling pathway may be activated.^44^ Nomura *et al*.^19^ also found that 22 of 26 GA‐FG lesions (84.6%) showed nuclear β‐catenin accumulation (LI: mean, 28.0%) and 10 of 26 GA‐FG lesions (38.5%) harbored at least one gene mutation of *CTNNB1, AXINs,* and *APC*. In addition, activating mutations in *GNAS* (p.R201C) were found in 5 of 26 GA‐FG lesions (19.2%). Notably, four of the five GA‐FG lesions with *GNAS* mutations showed nuclear β‐catenin accumulation, and the presence of *GNAS* mutations was associated with nuclear β‐catenin accumulation (*P* = 0.01).^19^

The sonic hedgehog (Shh) signaling pathway plays an important role in maintaining the proliferation and differentiation of CGA cells.^45^, ^46^ The detection of several proteins related to the Shh pathway revealed that the scores for Patched (Ptch), Smoothened (Smo), and Glioma‐associated oncogene 1 (Gli1) proteins, respectively, were significantly lower in GA‐FGs than in CGAs (mean: Ptch, 2.6 *vs* 4.1, *P* = 0.011; Smo, 3.4 *vs* 4.4, *P* = 0.024; Gli1, 2.3 *vs* 4.2, *P* = 0.002)^47^; additionally, there were no significant associations between nuclear β‐catenin accumulation and the expression of Ptch, Smo, or Gli1 proteins in GA‐FG, indicating that the Shh signaling pathway may be independently responsible for GA‐FG tumorigenesis.

## Treatment and follow‐up

Considering that most GA‐FG lesions are less invasive, 273 of 303 lesions (90.1%) were treated using endoscopic submucosal dissection (ESD) or endoscopic mucosal resection (EMR). Since GA‐FG originates from the deep layers of the gastric mucosa, the margins after ESD or EMR were positive in six lesions, leading to further surgical intervention. Notably, 21 of 303 lesions (6.9%) were removed by surgery because of the large tumor size, deep tumor invasion, lymphovascular invasion, or nodal metastasis; of these, 2 lesions without lymphovascular invasion and nodal metastasis were completely resected by non‐exposed endoscopic wall‐inversion surgery (NEWS) or combined laparoscopic and endoscopic approach for neoplasia with a non‐exposure technique (CLEAN‐NET), which is appropriate for cases with high surgical risks.^6^, ^48^ Polypectomy alone was performed in the other nine lesions; however, no tumor recurrences were observed during follow‐up (range, 6–39 months). A long‐term follow‐up (range, 0–107 months) of other lesions after treatment showed that only 1 lesion experienced local recurrence at month 45,^12^ whereas a long‐term follow‐up (range, 12–380 months) of 19 lesions before treatment showed that only 2 lesions without malignant features appeared slightly enlarged and elevated at months 120 and 380, respectively.^32^, ^49^ Similar to GA‐FG, GA‐FGM was primarily treated with ESD or EMR without recurrence during long‐term follow‐up (range, 1–97 months). However, during long‐term follow‐up before treatment, one GA‐FGM lesion showed a somewhat lobulated contour and a partly reddish surface with marked atypia in tumor glands and cells at month 60, suggesting that GA‐FGM has a higher malignant tendency than GA‐FG.^36^

## Conclusions

GA‐FGs have been increasingly reported in recent years, primarily attributed to the widespread eradication of *H. pylori* and advancements in public health, but the disease lacks well‐defined diagnostic and treatment guidelines. Therefore, a comprehensive summary of the characteristics of GA‐FG cases will assist clinicians in the accurate diagnosis and effective treatment of this disease.

GA‐FG, originating from the deep layers of the gastric mucosa and primarily consisting of chief cells or parietal cells, shows differentiation to the gastric fundic gland. GA‐FG is a unique tumor entity of unclear etiology and poorly understood pathogenesis; therefore, it is necessary to gather additional endoscopic data to identify the characteristics of GA‐FG, which depends on careful endoscopic screening, even in cases of gastric mucosal atrophy caused by *H. pylori*. The morphological characteristics of GA‐FG can be observed on WLI, but they are easily missed during diagnosis and misdiagnosis. M‐NBI combined with EUS can be helpful in the diagnosis of this tumor; however, it still has some limitations. Therefore, uniform diagnostic standards need to be established. Some cases present with submucosal invasion, lymphovascular invasion, or nodal metastasis, requiring surgical management; however, ESD alone or EMR alone is sufficient to cure this tumor in most cases. During a long‐term follow‐up of GA‐FG before and after treatment, no significant progress or recurrence was observed; therefore, the current curative criteria remain to be elucidated.

The review covers not only the endoscopic and histopathological characteristics of the disease but also its management of treatment and follow‐up. It is based on a comprehensive analysis of a substantial number of cases, making the results reliable. Clinicians can use the summarized characteristics to diagnose and treat this disease effectively, which holds significant clinical importance.
